# Supplementary figures and images for: Recombinant human soluble thrombomodulin is associated with attenuation of sepsis-induced renal impairment by inhibition of extracellular histone release
Source: PLoS One. 2020 Jan 23;15(1):e0228093. doi: 10.1371/journal.pone.0228093 (PMC6977725; doi:10.1371/journal.pone.0228093)

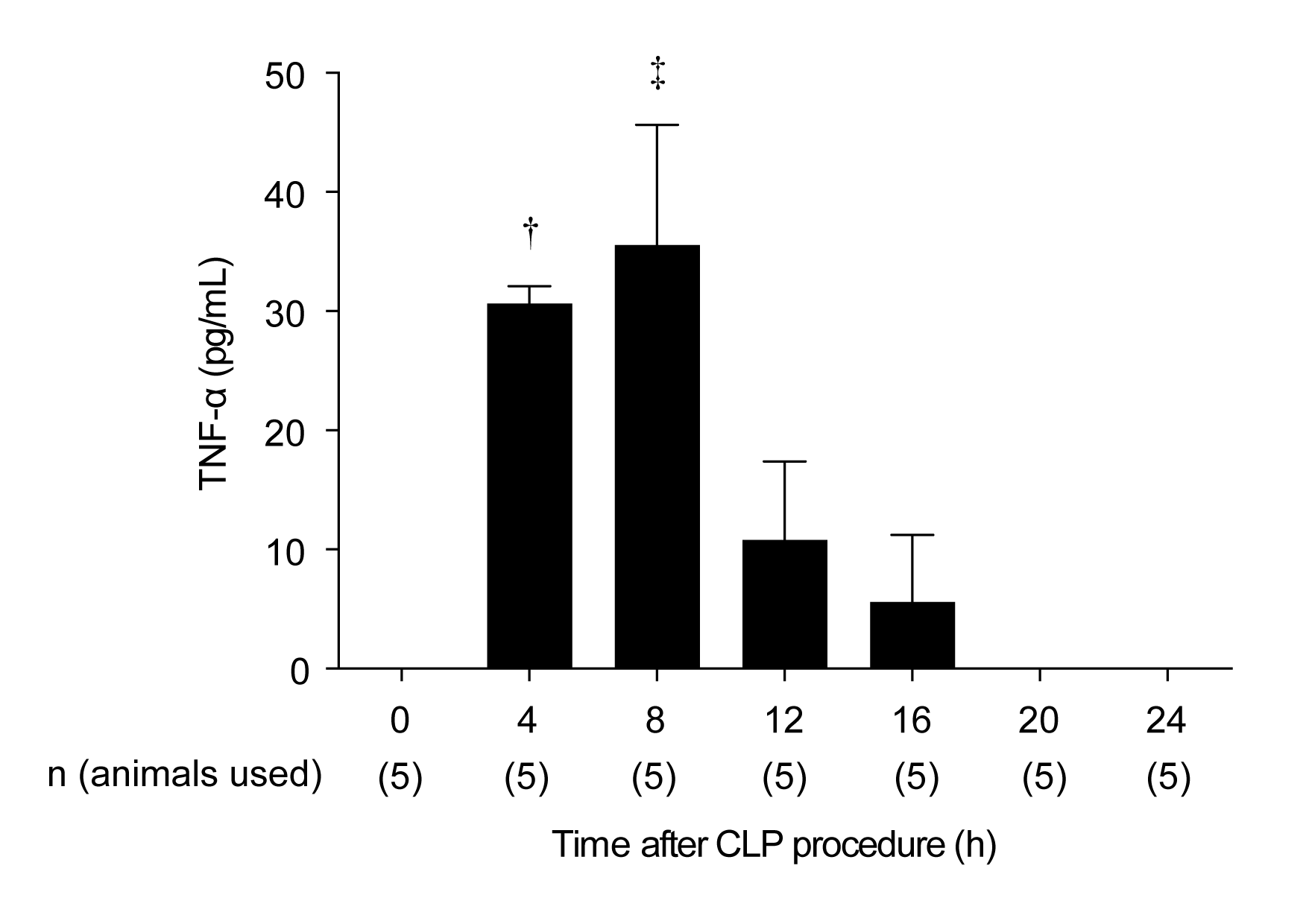

Supplement: S1 Fig — TNF-α level increased up to 8 h after the CLP procedure and then decreased over time. † P ≤ 0.01 vs. 0 h. ‡ P ≤ 0.001 vs. 0 h. (TIF) [file pone.0228093.s001.tif]
