# Supplementary material for: Recombinant human soluble thrombomodulin is associated with attenuation of sepsis-induced renal impairment by inhibition of extracellular histone release
Source: PLoS One. 2020 Jan 23;15(1):e0228093. doi: 10.1371/journal.pone.0228093 (PMC6977725; doi:10.1371/journal.pone.0228093)
Supplement: S1 Text — We investigated changes in TNF-α levels after CLP. TNF-α concentrations was measured using the rat TNF-α quantikine ELISA kit (R&D Systems, MN, USA) according to the manufacturer’s instructions. (DOCX) [file pone.0228093.s002.docx]

**Supporting Information Caption**

**S1 Text. Determination of TNF-α levels after CLP.** We investigated changes in TNF-α levels after CLP. TNF-α concentrations was measured using the rat TNF-α quantikine ELISA kit (R&D Systems, MN, USA) according to the manufacturer’s instructions.
